# Susceptibility to common insecticides and detoxifying enzyme activities in *Anopheles sundaicus* (*sensu lato*) after cessation of indoor residual spraying of insecticides in the Jaffna Peninsula and its surroundings in northern Sri Lanka

**DOI:** 10.1186/s13071-018-3254-1

**Published:** 2019-01-07

**Authors:** Kokila Sivabalakrishnan, Thilini C. Weerarathne, Amirtharasa Thileepan, S. H. P. Parakrama Karunaratne, Ranjan Ramasamy, Sinnathamby N. Surendran

**Affiliations:** 1 Department of Zoology, Faculty of Science, University of Jaffna, Jaffna, Sri Lanka; 2 Department of Zoology, Faculty of Science, University of Peradeniya, Peradeniya, Sri Lanka; 3 Office of the Provincial Director of Health Services, Jaffna, Sri Lanka; 4 ID-FISH Technology Inc., Palo Alto, California USA

**Keywords:** *Anopheles sundaicus* complex, Jaffna Peninsula, Insecticide-detoxifying enzymes, Insecticide resistance bioassays, Malaria, Mosquito vectors, Sri Lanka

## Abstract

**Background:**

Sri Lanka has been malaria-free since 2013 but re-introduction of malaria transmission by infected overseas travelers is possible due to a prevalence of potent malaria vectors. Knowledge of the insecticide resistance status among *Anopheles* vectors is important if vector control has to be reintroduced in the island. The present study investigated the insecticide susceptibility levels and resistance mechanisms of *Anopheles sundaicus* (*sensu lato*) (previously classified as *Anopheles subpictus* species B) an important malaria vector in the Jaffna Peninsula and it surroundings in northern Sri Lanka after indoor residual spraying of insecticides was terminated in 2013.

**Results:**

Species-specific PCR assays identified *An. sundaicus* (*s.l.*) in four locations in the Jaffna and adjacent Kilinochchi districts. Bioassays confirmed that *An. sundaicus* (*s.l.*) collected in Kilinochchi were completely susceptible to 0.05% deltamethrin and 5% malathion and resistant to 4% dichlorodiphenyltrichloroethane (DDT), whereas those from Jaffna were relatively susceptible to all three insecticides. Kilinochchi populations of *An. sundaicus* (*s.l.*) showed significantly higher glutathione S-transferase activity than population from Jaffna. However, Jaffna *An. sundaicus* (*s.l.*) had significantly higher Propoxur-resistant acetylcholinesterase activity. Activities of non-specific esterases and monooxygenases were not significantly elevated in *An. sundaicus* (*s.l.*) collected in both districts.

**Conclusions:**

The susceptibility to malathion and deltamethrin in *An. sundaicus* (*s.l.*) suggests that they can be still used for controlling this potential malaria vector in the Jaffna Peninsula and adjacent areas. Continuing country-wide studies on other malaria vectors and their insecticide susceptibilities are important in this regard.

## Background

Malaria had been endemic in Sri Lanka for centuries until indigenous transmission was eliminated from the island in 2013 [1]. However, many cases of malaria-infected travelers arriving from endemic countries are reported every year and therefore the potential for resuming indigenous transmission remains high due to the prevalence of many anopheline vectors in the island [1]. This challenge is exacerbated by the recent spread from India of the efficient urban malaria vector *Anopheles stephensi* to Sri Lanka [2, 3]. The Jaffna Peninsula in the Jaffna District and areas surrounding the Jaffna lagoon in the Kilinochchi District in northern Sri Lanka are coastal areas that were badly affected by malaria during the three decades of armed conflict that ended in 2009. The Anti-Malaria Campaign (AMC) in the north, specifically in the Kilinochchi District, faced logistic problems and a shortage of resources for its malaria control activities during the war.

*Anopheles culicifacies* species E was the major malaria vector in Sri Lanka while *An. annularis*, *An. subpictus* (*s.l.*) and *An. sundaicus* (*s.l.*) functioned as important secondary vectors together with other minor vectors before the elimination of malaria [4–10]. *Anopheles subpictus* exists as a species complex with members showing different bio-ecological traits relevant to malaria transmission [11, 12]. However, molecular genetic characterization of the *An. subpictus* complex after 2010 showed that mosquitoes previously identified morphologically as sibling species A, C and D belonged to a single group termed species A, while sibling species B belonged to the *An. sundaicus* complex [9, 10], a major vector of malaria in coastal zones of many Southeast Asian countries [13]. In Sri Lanka too *An. sundaicus* (*s.l.*) is mainly found in coastal zones [9, 10], which include the 1130 km^2^ Jaffna Peninsula and areas in the Jaffna and Kilinochchi districts that surround the Jaffna Lagoon [14].

Killinochchi and Jaffna districts in the Northern Province were among the previously malaria endemic administrative districts. The civil war of 1983–2009 in north and east of Sri Lanka limited studies on malaria vectors to a few in the Jaffna District. These studies suggested that morphologically characterized *An. subpictus* (*s.l.*) was the predominant anopheline species in Jaffna [15, 16] with a higher sporozoite rate than *An. culicifacies* [17]. The *An. sundaicus* (*s.l.*) identified at that time exclusively by morphology as *An. subpictus* species B, was the predominant anopheline species collected in 2008 from locations in the Jaffna District [15]. It was also found in 2008 that *An. subpictus* species B [now regarded as *An. sundaicus* (*s.l.*)] as well as *An. subpictus* species C and D (now regarded as *An. subpictus* A) were susceptible to 5% malathion but highly resistant to 4% dichlorodiphenyltrichloroethane (DDT) [15].

**Fig. 1:**
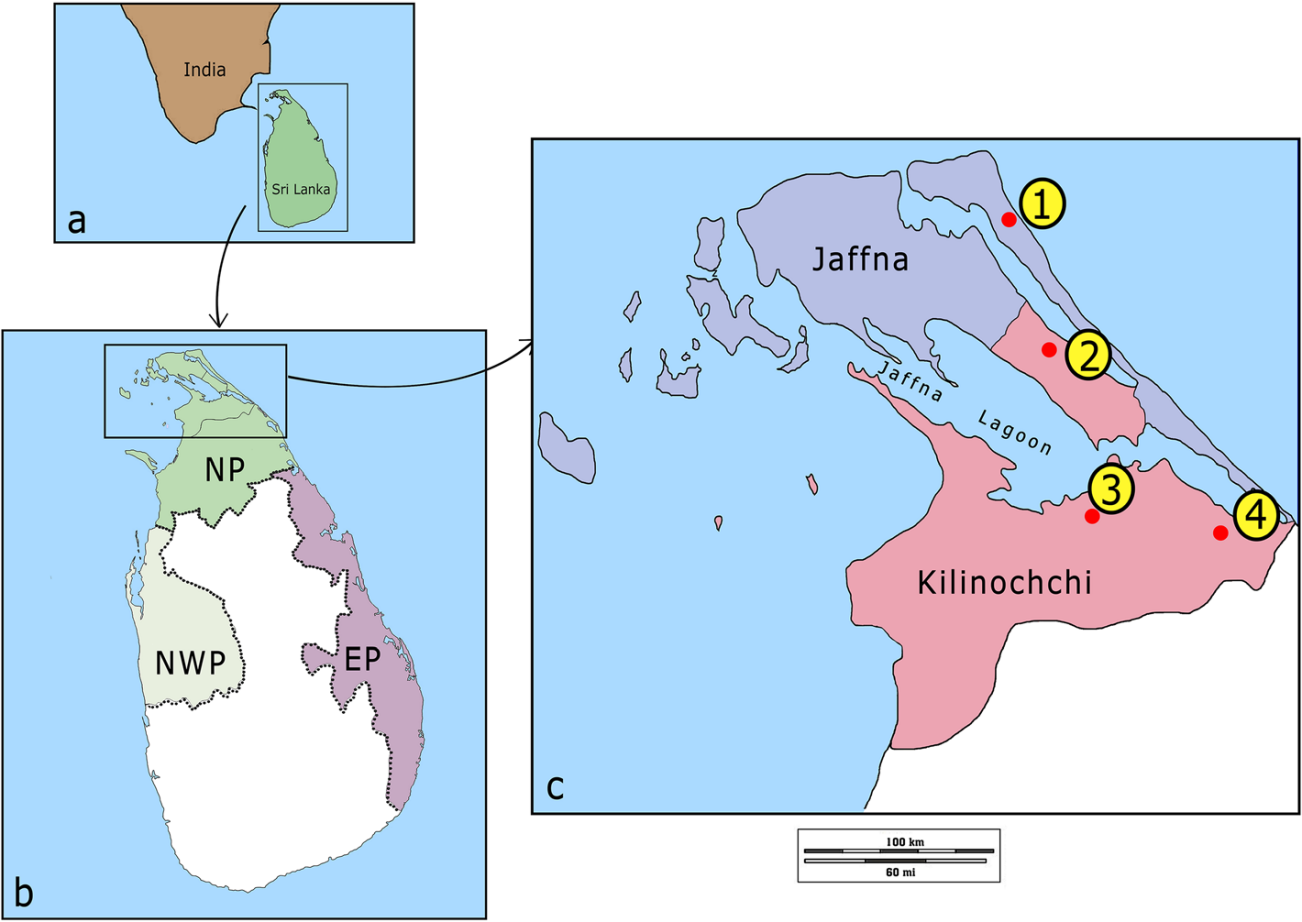
Locations of study sites. **a** Location of Sri Lanka in relation to India. **b** Administrative boundaries of Northern Province (NP), Eastern Province (EP) and North Western Province (NWP). **c** Locations of sample collection sites (1–4) in the districts of Jaffna and Kilinochchi

We previously reported insecticide susceptibility and resistance mechanisms in members of the *An. subpictus* complex, including sibling species B/*An. sundaicus* (*s.l.*), collected from sites in the North Western and Eastern provinces of Sri Lanka (Fig. 1) [18], but the study excluded the Northern Province because of the ongoing civil war. We have now extended this study to *An. sundaicus* (*s.l.*) collected from sites in the Jaffna Peninsula or its vicinity in the districts of Jaffna and Kilinochchi in the Northern Province. Understanding the insecticide resistance status and its biochemical basis in malaria vectors is important for vector control should indigenous malaria transmission re-emerge in the country.

## Methods

### Study locations, sample collection and species identification

Adult anopheline mosquitoes were collected monthly for 15months from December 2014 to February 2016 using cattle baited hut (CBH), cattle baited net (CBN) and indoor (IC) collection techniques [9, 10, 18] from three locations in the Kilinochchi District (Palai, Kandavalai and Iranaimadu) and one location (Kudathanai) in the Jaffna District (Fig. 1). These study sites were selected based on their previous malaria endemicity and coastal proximity. Each location had two or more mosquito collection points: 1. Kudathanai (3.4 km from the nearest coast) had four mosquito collection points (9°44'47.5332"N, 80°16'19.9956"E; 9°44'49.2828"N, 80°16'21.9288"E; 9°44'49.2432"N, 80°16'25.1328"E; and 9°44'44.9052"N,80°16'24.5532"E); 2. Palai (3 km from the nearest coast) had three mosquito collection points (9°36'26.838"N, 80°19'44.3892"E; 9°36'34.6032"N, 80°19'55.8228"E; and 9°36'20.4372"N, 80°19'51.0312"E); 3. Iranaimadu (16 km from the nearest coast) had two mosquito collection points (9°20'45.9708"N, 80°24'38.7432"E and 9°20'36.0636"N, 80°25'19.8408"E); and 4. Kandavalai (2 km from the nearest coast) had three mosquito collection points (9°27'14.1444"N, 80°29'9.7944"E; 9°27'38.2212"N, 80°29'13.1964"E; and 9°27'12.618"N, 80°29'38.5332"E).

Larval collections (LC) were carried out at each location from stagnant water bodies, e.g. sand pools and ponds, with 350 ml dippers and then reared as described previously under contained conditions in the insectary of the Department of Zoology, University of Jaffna to reach adulthood [18]. Larvae were maintained under laboratory conditions (28 ± 2 °C, 12h photoperiod and ~ 70% relative humidity) in the same water from the habitats where they were collected in 1.5 l capacity plastic trays with powdered fish meal given twice a day as additional food.

The collected anopheline mosquitoes and emerging adults from LC were identified morphologically as previously described [10, 18, 19]. Morphologically-identified blood-fed *An. subpictus* (*s.l.*) adults were maintained in the insectary to obtain F1 progeny as described previously [20]. Adults emerged from LC and F1 progeny of blood-fed *An. subpictus* (*s.l.*) mosquitoes were transferred to adult mosquito cages and fed on sugar pledgets. Other anopheline species identified in the collections were not processed for analysis. Three- to five-day-old *An. subpictus* (*s.l.*), the F1 progeny of field-collected adult females, as well as adults obtained from field-collected larvae were used for insecticide bioassays, biochemical assays and DNA-based identification.

### Bioassays for insecticide susceptibility

The standard World Health Organization (WHO) procedures were followed to determine the insecticide susceptibility status of adult mosquitoes [21]. Non-blood fed adult mosquitoes from the F1 progeny of field-collected blood-fed female mosquitoes and those developing from field-collected larvae in the Kilinochchi and Jaffna districts respectively, were pooled and separately tested for each district collection in duplicate assays with the WHO discriminating dosages of 0.05% deltamethrin, 5% malathion and 4% DDT using WHO bioassay test kits as previously described [18]. The WHO criteria were used to define a population as susceptible (> 98% mortality), suspected for resistance (90–98% mortality) and resistant (< 90% mortality) [22].

A total of 76 mosquitoes collected from the Jaffna (*n* = 37) and Kilinochchi (*n* = 39) districts identified morphologically were confirmed by PCR. This included 19 and 24 blood-fed females collected in the field at the sites in the Jaffna and Kilinochchi districts respectively that gave rise to F1 adult progeny used in the insecticide bioassay and enzyme assays in addition to adults that emerged from larval collections. A total of 397 and 305 adults derived from F1 progeny and field-collected larvae from Kilinochchi and Jaffna districts, respectively, were used for bioassays. These were made up of 66 and 90% of the F1 progeny of the adults that were used for species-specific PCR assays from Jaffna and Kilinochchi districts, respectively. Similarly, out of the 80 mosquitoes used for enzyme assays, 84 and 68% were from the F1 progeny of the blood-fed adults that were tested for species identification from sites in the Jaffna and Kilinochchi districts, respectively.

### Biochemical assays

Adult female mosquitoes emerging from F1 progeny and LC collections were subjected to biochemical assays using the microplate method described previously [18]. Eighty individuals from each district were subjected individually to total protein, carboxylesterase (EST), glutathione S-transferase (GST), monooxygenase (MO) and acetylcholinesterase (AChE) assays as described previously [18]. Specific activities of > 0.25 μmol/mg/min for EST, > 0.40 μmol/mg/min for GST, and > 0.35 equivalent units for MO were considered to be discriminating activity levels that can contribute to metabolic resistance in *An. subpictus* (*s.l.*) in Sri Lanka [18, 23]. According to WHO guidelines, remaining AChE activities after Propoxur inhibition in > 70%, 30–70% and < 30% of the population were categorized as homozygous resistant (RR), heterozygous resistant (RS) and homozygous susceptible (SS), respectively [24].

### Allele-specific PCR assay (AS-PCR) to distinguish *An. sundaicus* from *An. subpictus*

DNA from adult females that gave F1 progeny and individual mosquitoes emerging from field-collected larvae was extracted using Qiagen DNeasy Blood & Tissue Kit (Qiagen, Hilden, Germany) according to the manufacturer’s instructions. The extracted DNA was used for diagnostic allele specific PCR as described previously [10]. The diagnostic size of the PCR product for *An. subpictus* species A was ~300 bp while that for *An. sundaicus* (*s.l.*) was ~400 bp as previously reported [10].

### Data analysis

The two-tailed Student’s t-test for matched samples was performed to determine significant differences between the Kilinochchi and Jaffna mosquito populations in the susceptibility to insecticides and enzyme activities of GST, EST and MO. The Chi-square test was performed to assess significant differences in proportions of the three categories of AChE (SS, RS and RR) between mosquitoes collected in the two districts.

## Results

During the 15 month study period a total of 980 and 752 adults were morphologically identified as *An. subpictus* (*s.l.*) from collections in the Kilinochchi and Jaffna districts, respectively. *Anopheles annularis*, *An. barbirostis*, *An. culicifacies* (*s.l.*), *An. jamsai* and *An. psedojamsai* were present in the collections but not used in the studies. The collected *An. subpictus* (*s.l.*) from both districts collectively comprised 53, 25, 19 and 3% of mosquitoes collected by LC, CBN, CBH and IC, respectively.

Mosquitoes collected from Kilinochchi showed 100% mortality to both 0.05% Deltamethrin and 5% malathion and but only 31% mortality to 4% DDT indicating resistance to DDT. Although mosquitoes collected from Jaffna showed high mortality with deltamethrin (97%), malathion (96%) and DDT (91%), the results indicate the possibility of some resistance to all three insecticides according to the WHO criteria [21, 22]. The Kilinochchi population showed significantly higher resistance to DDT than Jaffna population, but there were no statistically significant differences in susceptibility to deltamethrin and malathion between the two districts (Table 1).

**Table 1:** Mortality in *An. sundaicus* (*s.l.*) exposed to three insecticides

Insecticide	Mean % mortality ± SD (no. of mosquitoes tested)		*t*-value	*P*-value
	Kilinochchi	Jaffna		
Deltamethrin (0.05%)	^S^100 (110)	^V^97.3 ± 3.8 (100)	*t*_(8)_=-3.0	0.09
Malathion (5%)	^S^100 (136)	^V^96. 4 ± 0.7 (105)	*t*_(10)_= -2.0	0.18
DDT (4%)	^R^30.9 ± 8.7 (151)	^V^91.2 ±0.3 (100)	*t*_(8)_= 31.3	0.01

*Abbreviations*: *S* susceptible (≥ 98% mortality), *R* confirmed resistance (< 90% mortality), *V* possible resistance and verification needed (90–97% mortality) [22], *SD* standard deviation

The enzyme activities and the percentage of mosquito populations that had enhanced enzyme activities are shown in Table 2. Although significantly different activities of EST (*t*_(155)_=3.76, *P* < 0.001) and MO (*t*_(150)_= 15.53, *P* < 0.001) were observed between the Kilinochchi and Jaffna population, neither population alone or collectively had activities of the two enzymes above the discriminatory levels for resistance reported for Sri Lankan *An. subpictus* (*s.l.*) [23]. Significantly (*t*_(148)_= -16.98, *P* < 0.001) elevated GST activities above the reported discriminatory levels were seen in all of the Kilinochchi population compared with only 30% of the Jaffna population. The results of AChE assays to detect the percentage remaining activityof AChE in the presence of Propoxur are also presented in Table 2. The Chi-square test revealed a significant (*χ*^2^= 13.41, *P* = 0.0012) association between the districts and the remaining activity of AChE in the three WHO categories of resistance, with the Jaffna *An. sundaicus* (*s.l.*) mosquitoes showing a greaterAChE active site alteration than in Kilinochchi.

**Table 2:** *Anopheles sundaicus* (*s.l.*) populations with discriminating activities of GST, EST and MO and insensitive target site AChE

District	Enzyme activity profiles	AChE (%)^a^			GST^b^	EST^c^	MO^d^
		< 30 [SS]	30–70 [RS]	> 70 [RR]			
Jaffna district	% population(a) in different categories and (b-d) with elevated activities	50	35	15	30	0	0
	Mean activity ± SE				0.38 ±0.02	0.04 ±0.002	0.03 ±0.001
Kilinochchi district	% population(a) in different categories and (b-d) with elevated activities	78	17	5	100	0	0
	Mean activity ± SE				1.85 ±0.08	0.06 ±0.004	0.003 ±0.001
Pooled data for both districts	% population (a) in different categories and (b-d) with elevated activities	68	23	9	69	0	0
	Mean activity ± SE				1.14 ±0.09	0.06 ±0.004	0.02 ±0.002

*Abbreviation*: *SE* standard error of the mean

^a^Percent remaining activity of AChEs in individual mosquitoes after Propoxure inhibition in homozygous (SS) sensitive, heterozygous (RS) and homozygous (RR) insensitive populations [24]

^b^Percentage of population having glutathione S-transferase (GST) discriminant specific activity above 0.40 μmol/mg/min and mean specific activities

^c^Percentage of population having esterase (EST) discriminant specific activity above 0.25 μmol/mg/min and mean specific activities

^d^Percentage of population having monooxygenase (MO) levels above the discriminant activity of 0.35 units per mg protein of cytochrome P_450_ and mean specific activities [18, 23]

## Discussion

**Fig. 2:**
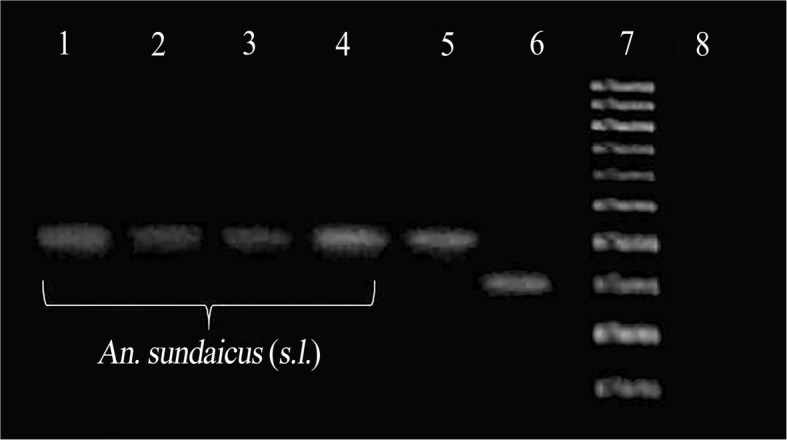
Agarose gel image of species-specific PCR assay to identify *An. sundaicus* complex from *An. subpictus* complex. The diagnostic size of the PCR product is ~ 400 bp for *An. sundaicus* complex. Lanes 1–4: *An. sundaicus* (*s.l.*) samples; Lane 5: *An. sundaicus* (*s.l.*) positive control from a previous study [10]; Lane 6: *An. subpictus* species A control from previous study [10]; Lane 7: 100 bp DNA ladder as size marker; Lane 8: negative PCR control without added DNA

Because the PCR assays (Fig. 2) revealed that all 76 tested specimens belonged to *An. sundaicus* (*s.l.*), and all *An. subpictus* species B-like mosquitoes recently independently identified through existing morphological criteria in coastal and inland northern Sri Lanka were shown genetically belong to the *An. sundaicus* complex [9, 10], it is reasonable to assume that the vast majority, if not all the mosquitoes tested in the insecticide bioassay and enzymatic assays, are *An. sundaicus* (*s.l.*) and not *An. subpictus* species A. To our knowledge, the present study is the first to investigate insecticide resistance and insecticide resistance mechanisms in *An. sundaicus* (*s.l.*) in the Northern Province of Sri Lanka.

The greater resistance to DDT of mosquitoes from Kilinochchi compared to Jaffna may be due the higher prevalence of elevated GST activity in Kilinochchi compared with Jaffna. DDT resistance in morphologically identified *An. subpictus* (*s.l.*) was first reported in 1969 and a reduction in resistance was detected after cessation of DDT use for IRS and its replacement with malathion in the early 1970s [1, 25]. Later, due to the development of a GST-based resistance mechanism, which was suggested to be favored by high DDT application prior to malathion introduction, an increase in the DDT-resistant population was observed among *An. subpictus* complex [suspected to be a mixture of *An. subpictus* species A and *An. sundaicus* (*s.l.*)] after 1983 [26, 27]. No elevation of EST or MO was detectable in the two populations, suggesting that that EST and MO do not contribute to DDT resistance in the two districts.

*Anopheles sundaicus* (*s.l.*) populations, except that of Northern Province, are reported to have developed resistance to pyrethroid insecticides in other parts of the country [23]. Perhaps the relatively limited previous use of pyrethroids for vector control in the two northern districts for IRS and the absence of elevated ES and MO might be the reason for the observed relative susceptibility to deltamethrin. The results suggest that the Jaffna *An. sundaicus* (*s.l.*) mosquitoes may show weak resistance to deltamethrin but confirming this and the investigating potential underlying mechanisms requires more extensive investigation. The higher percentage remaining activity of AChE seen in the Jaffna population may be due to a continuing and more intensive use of organophosphate and carbamates pesticides for agriculture in the Jaffna Peninsula. Further studies with more sampling sites from both districts are needed to establish this.

Indoor residual spraying (IRS) was, until recently, the principal method of malaria vector control in Sri Lanka. Sri Lanka has undertaken different insecticide regimes at different times over the last six decades for malaria control [18, 23]. DDT introduced at the end of World War 2 for IRS was highly effective in controlling malaria until resistance developed in the 1960s and 1970s, causing it to be replaced by the organophosphate malathion in 1977. Pyrethroids have been used for IRS since 1994 on the whole island except for the Northern Province due to the development of resistance to malathion [1]. However, IRS has been scaled down or has ceased since 2013 in the island and is now only performed in the vicinity of the residences of persons identified to have contracted malaria abroad.

In northern Sri Lanka, malaria control activities were curtailed in the Jaffna and Kilinochchi districts during the civil war as the regional AMC faced logistical problems and a shortage of resources. Vector control activities in the Jaffna and Kilinochchi districts in the Northern Province were mainly restricted to IRS with malathion until 2002 when it was replaced by the pyrethroid deltamethrin [15].

High susceptibility to common insecticides shown by *An. sundaicus *(*s.l.*) populations from other parts of the country was attributed to its exophagic and exophilic nature [18]. However, collection of *An. sundaicus* (*s.l.*) in IC and CBH techniques during the present and an earlier study [15], along with high sporozoite rates [17], indicates some endophagic and endophilic behavior in northern Sri Lanka, for which IRS and insecticide-treated bed nets can be effective. Our previous study on the resistance and resistance mechanisms in Eastern and North Western provinces in coastal areas of mainland Sri Lanka (Fig. 1) suggested that *An. sundaicus* (*s.l.*) populations were resistant to DDT but relatively susceptible to malathion and pyrethroids [18], compatible with the present observations in Northern Province. Cessation of malathion use for IRS since 1994 on the whole island and in Northern Province in 2002, along with the careful use of pyrethroids for IRS and its cessation in 2006, may contribute to the continued relative susceptibility to the two insecticides. Development of resistance is associated with a fitness cost and mosquito populations can in time lose resistance in the absence of insecticide selection pressure [28, 29]. It is pertinent, however, that organophosphate and pyrethroid insecticides continue to be used for agricultural purposes and personal protection, respectively, in Sri Lanka [30], and this might eventually contribute to the development of resistance to the two classes of insecticides.

## Conclusions

The results suggest that malathion and deltamethrin may still be effectively used to control *Anopheles sundaicus* (*s.l.*) in the Jaffna and Kilinochchi districts but indicate the need for continued monitoring of insecticide resistance in the two districts and elsewhere in the country.

## Abbreviations

AChE: Acetylcholinesterase
AMC: Anti-Malaria Campaign
DDT: Dichlorodiphenyltrichloroethane
EST: Esterase
GST: Glutathione S-transferase
IRS: Indoor residual spraying
MO: Monooxygenase
PCR: Polymerase chain reaction
